# Characterization of recent and minimally passaged Brazilian dengue viruses inducing robust infection in rhesus macaques

**DOI:** 10.1371/journal.pone.0196311

**Published:** 2018-04-25

**Authors:** Maria Beatriz Borges, Renato Sergio Marchevsky, Ygara S. Mendes, Luiz Gustavo Mendes, Ana Claudia Duarte, Michael Cruz, Ana Maria Bispo de Filippis, Pedro Fernando C. Vasconcelos, Marcos Freire, Akira Homma, Sally Mossman, Edith Lepine, Yannick Vanloubbeeck, Clarisse Lorin, Marie-Pierre Malice, Elena Caride, Lucile Warter

**Affiliations:** 1 Fiocruz, Rio de Janeiro, Brazil; 2 GSK, Rockville, Maryland, United States of America; 3 Instituto Evandro Chagas, MoH, Ananindeua, Brazil; 4 GSK, Rixensart, Belgium; CEA, FRANCE

## Abstract

The macaque is widely accepted as a suitable model for preclinical characterization of dengue vaccine candidates. However, the only vaccine for which both preclinical and clinical efficacy results were reported so far showed efficacy levels that were substantially different between macaques and humans. We hypothesized that this model’s predictive capacity may be improved using recent and minimally passaged dengue virus isolates, and by assessing vaccine efficacy by characterizing not only the post-dengue virus challenge viremia/RNAemia but also the associated-cytokine profile. Ten recent and minimally passaged Brazilian clinical isolates from the four dengue virus serotypes were tested for their infectivity in rhesus macaques. For the strains showing robust replication capacity, the associated-changes in soluble mediator levels, and the elicited dengue virus-neutralizing antibody responses, were also characterized. Three isolates from dengue virus serotypes 1, 2 and 4 induced viremia of high magnitude and longer duration relative to previously reported viremia kinetics in this model, and robust dengue virus-neutralizing antibody responses. Consistent with observations in humans, increased MCP-1, IFN-γ and VEGF-A levels, and transiently decreased IL-8 levels were detected after infection with the selected isolates. These results may contribute to establishing a dengue macaque model showing a higher predictability for vaccine efficacy in humans.

## Introduction

Dengue is the most widespread arboviral disease affecting humans. It is caused by dengue virus (DENV), an enveloped virus with a positive single-stranded RNA genome belonging to the *Flaviviridae* family. There are four DENV serotypes (DENV-1 to DENV-4) that can all cause clinical manifestations in humans ranging from mild to life-threatening severe dengue [1]. While the global annual incidence has been estimated at 50–100 million symptomatic dengue cases [2], no DENV-specific therapeutics are available, and the only licensed vaccine, Dengvaxia, has shown variable efficacy depending on the infecting DENV serotype and age of the recipient [3,4]. This necessitates development of improved DENV-specific vaccine(s).

DENV-related research has been impaired by the lack of an immunocompetent animal model reproducing human dengue disease. Although several monkey species (including rhesus and cynomolgus macaques) sustain DENV replication after experimental infection, they rarely develop clinical symptoms [5–7]. Despite this, the macaque is the most widely accepted model for preclinical characterization of DENV-specific vaccine candidates which were, prior to their clinical development, all tested for efficacy in this model. In these studies, vaccinated macaques were subcutaneously challenged with DENV and post-challenge viral replication was measured as a surrogate of disease [8–13]. However, Dengvaxia, the only DENV vaccine for which both preclinical and clinical efficacy results were reported so far showed almost 100% efficacy at preventing post-challenge viremia in macaques whereas its overall efficacy in humans was substantially lower (56.5% and 60.8% in Asia and Latin America, respectively) [3,4,11]. Although this discrepancy might be attributed to possible differences in the vaccine lots tested in preclinical and clinical studies, it may also cause one to question the relevance of the dengue macaque model as it currently exists.

One possible explanation for the limited predictability of this model may be that the viremia levels are substantially lower in macaques when compared with those detected during clinically apparent infections in humans [5,14,15]. Therefore, protection from low-level viremia in macaques may not predict protection from dengue in humans.

Most DENV strains used as challenge viruses were isolated many years ago and subjected to multiple sequential passages within the same cell culture system [8–13]. Importantly, while arboviruses in the wild have shown high levels of nucleotide sequence conservation over time [16,17], the mutation rate increases drastically when the host alteration is bypassed, such as when these viruses are passaged serially in a single cell type or in the same host [16,18]. Therefore, DENV strains that have been sequentially passaged in the same cell culture system may differ significantly from circulating DENV strains, and protection from such cell-passaged viruses might not predict protection from natural infection.

Several soluble mediators are believed to play a key role in the increased vascular permeability leading to plasma leakage and coagulopathy, the hallmarks of severe dengue in humans [1,19,20]. The factors most frequently described as showing modified levels during dengue fever/severe dengue include the pro-inflammatory cytokines interleukin (IL)-2, IL-6, IL-8, interferon (IFN)-γ and tumor necrosis factor (TNF)-α, the anti-inflammatory cytokine IL-10, the chemokines macrophage inflammatory protein (MIP)-1α, MIP-1β, and monocyte chemoattractant protein (MCP)-1, and the vascular endothelial growth factor (VEGF)-A [1,19–26]. Provided that some of these cytokines are shown to be similarly associated with DENV infection in macaques, combining their characterization with measurement of post-challenge viral replication could improve the predictability to humans of efficacy results obtained in the dengue macaque model.

To improve the dengue macaque model, we selected minimally passaged DENV clinical isolates that robustly replicate in rhesus macaques, and characterized the associated changes in soluble cytokine levels. Ten Brazilian DENV clinical isolates were tested for their replication capacity in rhesus macaques and fifteen mediators, previously reported as showing modified levels in humans experiencing dengue fever/severe dengue, were tested for their serum concentration after DENV inoculation. Three DENV clinical isolates were identified as inducing viremia of long duration and high magnitude relative to previously reported viremia kinetics in this model [5,27]. After DENV infection, four soluble mediators were detected as showing modified levels similar to those reported in humans. Taken together, these results may help in the development of a dengue macaque model of higher predictability for the response to dengue vaccines in humans.

## Materials and methods

### Cell culture and viruses

Vero cells (ATCC No CCL-81) were grown at 37°C in a humidified 5%-CO_2_ incubator in medium 199 with Earle's salts supplemented with 10%-FBS (Gibco). The strains tested in monkeys are described in Table 1. Strains 0111/2011, 0126/2010, 0498/2010, 2935/2013 and 1071/2012 were obtained from Instituto Oswaldo Cruz (Rio de Janeiro, Brazil) and strains MÃO 9487, ROR 6210, BEL 74561, BEL 83791 and ROR 7591 from Instituto Evandro Chagas (Ananideua, Brazil). The DENV-1 60305 [28], DENV-2 44/2 [29], DENV-3 16562 [28] and DENV-4 TVP360 strains were used in the plaque-reduction neutralization test (PRNT).

**Table 1:** Characteristics of the DENV strains tested for their infectivity in rhesus macaques.

DENV serotype	DENV isolate	Isolation site	Isolation year	Associated clinical course	Cell passage history^a^	Master viral stock titer		Working viral stock titer	
						log_10_ PFU or FFU/mL^b^	log_10_ eg/mL^c^	log_10_ PFU or FFU/mL^b^	log_10_ eg/mL^c^
**DENV-1**	**0111/2011**	Rio de Janeiro, Brazil	2011	SD	**3 CP** (1x C6/36 and 2x Vero)	6.3	9.1	7.0	9.1
**DENV-2**	**0126/2010**	Rio de Janeiro, Brazil	2010	SD	**3 CP** (1x C6/36 and 2x Vero)	6.3	8.0	6.7	7.7
**DENV-3**	**0498/2010**	Rio de Janeiro, Brazil	2010	DF	**4 CP** (2x C6/36 and 2x Vero)	5.0	7.2	7.1	8.0
	**BEL 74561**	**Belém, Brazil**	2004	DF	**4 CP** (including 2x Vero)	6.3	5.7	7.7	5.9
	**ROR 6210**	**Roraima, Brazil**	2007	DF	**4 CP** (including 2x Vero)	5.2	5.8	8.0	5.5
	**MÃO 9487**	**Maranhao, Brazil**	2002	DF	**4 CP** (including 2x Vero)	5.5	6.7	6.9	7.8
**DENV-4**	**2935/2013**	Rio de Janeiro, Brazil	2013	DF	**4 CP** (2x C6/36 and 2x Vero)	7.5	10.2	7.2	8.1
	**ROR 7591**	**Roraima, Brazil**	2010	DF	**4 CP** (including 2x Vero)	7.5	7.9	8.4	6.5
	**1071/2012**	**Rio de Janeiro, Brazil**	2012	SD	**3 CP** (1x C6/36 and 2x Vero)	6.3	10.1	7.4	8.2
	**BEL 83791**	**Belém, Brazil**	2011	DF	**4 CP** (including 2x Vero)	7.5	7.8	7.5	6.7

^a^ Total number of cell passages including those performed for isolation and production of master and working viral stocks

^b^ As measured by plaque or focus assay (DENV-1, DENV-2 and DENV-3, DENV-4, respectively) depending on the plaque-forming capacity of the tested strains

^c^ As measured by DENV-specific real-time RT-PCR.

CP, cell passage; DF, dengue fever; SD, severe dengue.

### Infection of monkeys and blood collection

The study protocol was approved by the Institutional Ethical Committee for Use of Animals (CEUA-Fiocruz) and conducted in strict accordance with the recommendations from the Guide for Care and Use of Laboratory Animals of the Brazilian Society of Science in Laboratory Animals and the National Council for the Control of Animal Experimentation. Thirty male adult rhesus macaques (*Macaca mulatta*) of Indian origin, flavivirus-naive and colony-born in captivity in the Non-human Primates Breeding Service from the Institute of Science and Technology in Biomodels of the Oswaldo Cruz Foundation (Rio de Janeiro, Brazil), were used in this study. The experiment was performed in a biohazard level 2 animal facility (temperature 20–22°C; humidity 50–60%; light/dark cycle 12 h/12 h). Monkeys were acclimated for 14 days before study start. Monkeys were housed individually but retained in a social environment through visual contact with other monkeys. Polished stainless steel mirrors, PVC and wooden teethers, as well as foraging tray containing food such as pieces of cereal bars, raisins, rice grains or sunflower seeds were given as environmental enrichment. Monkeys had free access to water and received a commercial diet (Nuvilab Primates 6030 Nuvital) supplemented with fresh fruits and vegetables. Throughout the study, monkeys were observed twice a day by animal care and veterinary staff for health and well-being assessment. None of the monkeys became ill or died prior to the end of the study. Monkeys were anesthetized with ketamine (8–10 mg/kg) prior to virus inoculation and blood drawing. For virus inoculation, 0.5 mL of sterile culture medium containing 10^5^ plaque- or focus-forming units (PFU and FFU, respectively) (a DENV dose previously shown to induce viremia in rhesus macaques and within the range of 10^4^−10^5^ PFU expected to be transmitted by *Aedes spp*. [6,30]) were administered subcutaneously. After inoculation, titer of the residual viral inoculum was confirmed by plaque or focus assay. At the end of the study, monkeys were anesthetized by intra-muscular injection of ketamine (20 mg/kg) prior to being euthanized by intra-peritoneal injection of thiopental sodium (50 mg/kg).

### Virus titration by plaque/focus assay

Serial dilutions of viral stocks or macaque sera were added to Vero cells previously seeded into 6-well plates. After 1 h at 37°C, the diluted samples were replaced by maintenance medium supplemented with 3%-carboxyl-methyl-cellulose (CMC). For plaque assays (DENV-1 and DENV-2 strains), eight days later cells were fixed overnight with a 10%-formaldehyde solution prior to crystal violet staining. For focus assays (DENV-3 and DENV-4 strains), eight days later cells were fixed for 1 h with a 4%-formaldehyde solution prior to detecting DENV-infected cells using 7 μg/mL HRP-conjugated pan-flavivirus 4G2 monoclonal antibody (in-house production) followed by incubation with TrueBlue (KPL, Gaithersburg, MD). PFU/FFU were counted by the naked eye and infectious virus titers were expressed as PFU or FFU/mL.

### DENV genome equivalents quantification by real-time RT-PCR

Viral RNA was extracted from 200 μL of either cell culture supernatant or monkey sera using the High Pure Viral Nucleic Acid kit (Roche). DENV genome equivalents were quantitated by real-time RT-PCR using the AgPath-ID One-Step RT-PCR kit (Ambion). Each RT-PCR reaction mixture contained 2.5 μl of RNA, 1.67 μL of Detection Enhancer, 2X RT-PCR Buffer, 25X RT-PCR Enzyme Mix (all from the kit), 20 U of RNAsin (Ambion), as well as 10 and 5 pmol of DENV serotype-specific forward/reverse primers and probes, respectively. Primers and probes used were: DENV1, forward 5’-GCA-TTY-CTA-AGA-TTT-CTA-GCC-ATA-CC-3’, reverse 5’-TCG-CTC-CAT-TCT-TCT-TGA-ATG-AG-3’, probe 5’-AAC-AGC-AGG-AAT-TTT-3’; DENV2, forward 5’-CTG-CAR-GGA-CGA-GGA-CCA-TT-3’, reverse 5’-GGG-ATT-GTT-AGG-AAA-CGA-AGG-A-3’, probe 5’-AAA-CTG-TTC-ATG-GCC-CTG-GTG-GCR-3’; DENV3, forward 5’ TCGCTCTGTCTCATGATGATRTT-3’, reverse 5’- GGCTCTCCATCGCGTGAA-3’, probe 5’-CCAGCAACACTTGCTTTCCACTT-3’; DENV4, forward 5’-TCTCTGGAAAAATGAACCAACGA-3’, reverse 5’- CGGTTTCTCTCGCGTTTCAG-3’, probe 5’-AAAAGGTGGTTAGACCACCTTTCAATAT-3’. An *in vitro* transcribed DENV RNA, quantitated by optical density measurement, was used as a standard. RT was performed using an ABI 7500 Real-Time PCR system (Applied Biosystems) at 45°C for 10 min, followed by an incubation step at 95°C for 10 min and 40 cycles of 15 sec at 95°C and 1 min at 60°C.

### Plaque-reduction neutralization test (PRNT)

100 DENV PFU were mixed with equal volumes of serially diluted sera and incubated for 1 h at 37°C. The mixtures were added for 1 h at 37°C onto Vero cells previously seeded into 6-well plates, and subsequently replaced by maintenance medium supplemented with 3%-CMC. Seven (DENV-2) or eight (DENV-1, DENV-3 and DENV-4) days later, cells were fixed overnight with 10%-formaldehyde solution prior to crystal violet staining. PFU were counted by the naked eye and the percent neutralization was determined relative to the number of PFU counted with the virus control (corresponding to 0% neutralization). PRNT50 titers, corresponding to the reciprocal serum dilution associated with 50% reduction in plaque counts, were determined using a linear model.

### Cytokines quantification

Undiluted sera were tested in duplicate using the MILLIPLEX MAP Non-Human Primate Cytokines Magnetic Bead Panel Kit (Merck). Data were acquired and analyzed using the Luminex 200 reader (Merck). Results were expressed as pg/mL. Statistical analysis was based on an ANCOVA model for the change from baseline with fixed effect for strain, day, and including the baseline value as covariate. The covariance matrix for the repeated measures across days was assumed to be of the Toeplitz type. In case of non-convergence of the model, a compound symmetry structure was assumed. The model assumed equal variance across strains.

## Results

### Selection of recent and low-passage DENV strains from serotypes 1, 2 and 4 inducing high viral replication in rhesus macaques

A large panel of Brazilian DENV isolates was first selected based on two criteria: being recently isolated from human patients and not passaged more than twice in cell culture. The isolates were amplified through two additional passages in Vero cells to produce viral stocks which were tested for their DENV titer after each amplification step. Isolates to be further tested for their infectivity in macaques were selected based on two technical criteria: final viral stock titer ≥ 10^6^ PFU or FFU/mL (to allow *in vivo* inoculation of ~10^5^ PFU or FFU/0.5 mL) and consistent titers between the two amplification steps (assuming that a drastic increase in viral titer might indicate a virus adaptation to the cell culture and thus loss of wild-type characteristics). The DENV isolates selected for further *in vivo* evaluation are described in Table 1.

Based on previously reported DENV viremia peak levels in rhesus macaques, ranging from 10^1.6^ to 10^3.6^ PFU/mL depending on serotype and strain [5], success criteria for robust infectivity were defined as at least two consecutive days of viremia with a peak ≥ 100 PFU or FFU/mL in 100% of the inoculated macaques. Each isolate was first inoculated into two macaques in parallel. The DENV replication was monitored by measuring viremia and RNAemia in sera collected daily after inoculation. If an isolate met the success criteria, its infectivity was further confirmed in at least 3 additional macaques. In contrast, if robust infectivity was not demonstrated, the corresponding isolate was excluded and alternative isolates from the same serotype were assessed for their replication capacity.

**Fig 1:**
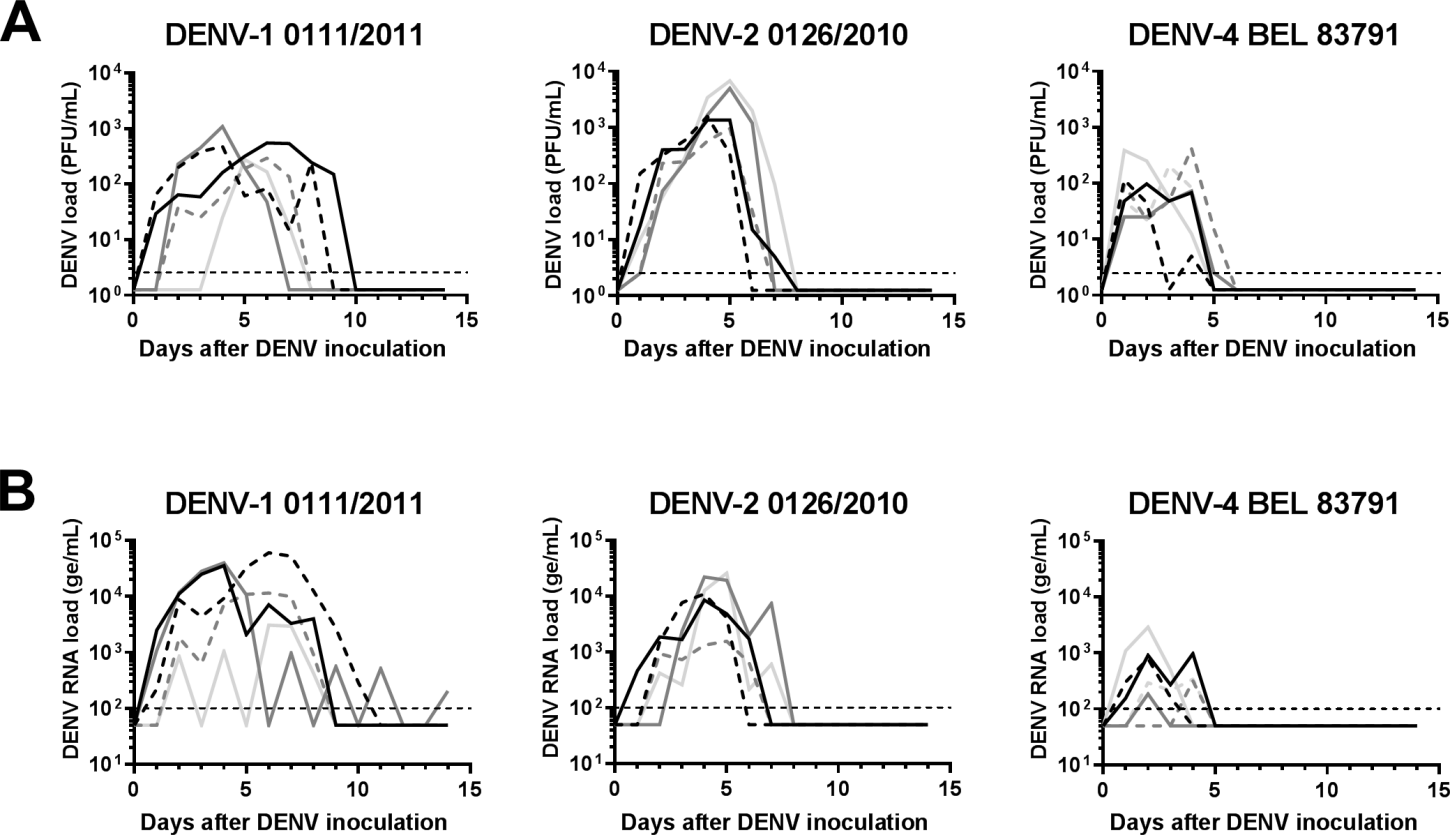
Viremia and RNAemia detected after inoculation with DENV-1 0111/2011, DENV-2 0126/2010 or DENV-4 BEL 83791. Rhesus macaques were subcutaneously inoculated with ~10^5^ plaque- or focus-forming units (PFU and FFU, respectively) of DENV-1 0111/2011 (n = 5), DENV-2 0126/2010 (n = 5) or DENV-4 BEL 83791 (n = 6). Sera were collected daily during the 14 days post-inoculation and tested, in duplicate, by plaque or focus assay for their infectious virus content, *i*.*e*. viremia, expressed as PFU or FFU/mL (A) and by real-time RT-PCR for their DENV genome equivalent (ge) content, *i*.*e*. RNAemia, expressed as ge/mL (B). The individual viremia and RNAemia curves are shown. Horizontal dashed lines indicate the threshold of detection for the plaque/focus assay and the real-time RT-PCR assay, i.e. 2.5 PFU or FFU/mL and 10^2^ ge/mL, respectively. In the absence of viremia and/or RNAemia detection, the corresponding sample was assigned an arbitrary titer corresponding to half the limit of detection.

Viremia and RNAemia times to detection, durations and peaks are shown in Table 2. None of the four tested DENV-3 isolates met the pre-defined success criteria as two out of the four DENV-3 isolates did not induce any detectable viremia or RNAemia while the other two DENV-3 isolates induced only short and low-level viremia and RNAemia. The only DENV-1 and DENV-2 isolates tested, DENV-1 0111/2011 and DENV-2 0126/2016, fully met the success criteria as yielding mean duration and peak of viremia of 6.4 days and 10^2.73^ PFU/mL and 6.2 days and 10^3.50^ PFU/mL, respectively (Table 2 and Fig 1). Four different DENV-4 isolates were tested. Of these, DENV-4 2935/2013 induced short and low viremia and was thus excluded from further testing. Two other DENV-4 isolates, DENV-4 ROR 7591 and DENV-4 1071/2012, although resulting in mean durations and peaks of viremia of 4 days and 10^2.52^ FFU/mL and 5.5 days and 10^1.84^ FFU/mL, respectively, were not further tested *in vivo* as the criteria for robust replication were met in only one out of the two inoculated macaques. In contrast, the last DENV-4 isolate that was tested, DENV-4 BEL 83791, met the criteria for robust infectivity in all inoculated macaques, with a mean duration and peak of viremia of 6.4 days and 10^2.73^ FFU/mL (Table 2 and Fig 1).

**Table 2:** Viremia and RNAemia as detected after DENV inoculation into rhesus macaques.

DENV serotype	DENV isolate	Number of inoculated animals	Viremia^a^			RNAemia^b^		
			Time to detection^c^	Duration^d^	Peak titer^e^	Time to detection^c^	Duration^d^	Peak titer^e^
**DENV-1**	**0111/2011**	5	2.0	6.4	2.7	1.2	8.6	4.5
**DENV-2**	**0126/2010**	5	1.0	6.2	3.5	2.0	5,2	4.1
**DENV-3**	**0498/2010**	2	2.0	3.5	1.7	2.0^f^	4.0^f^	2.7^f^
	**BEL 74561**	2	nd	nd	nd	nd	nd	nd
	**ROR 6210**	2	nd	nd	nd	nd	nd	nd
	**MÃO 9487**	2	2.5	1.5	1.4	2.0^f^	3.0^f^	1.9^f^
**DENV-4**	**2935/2013**	2	2.0^f^	1.0^f^	0.9^f^	1.0	3.0	3.2
	**ROR 7591**	2	1.0	4.0	2.5	2.5	5.5	3.5
	**1071/2012**	2	1.0	5.5	1.8	1.5	3.5	2.7
	**BEL 83791**	6	1.0	4.2	2.4	1.8	2.5	3.0

^a^Viremia was measured by plaque or focus assay and expressed as log10 plaque-forming units (PFU)/mL for DENV-1 and DENV-2 and log10 focus-forming units (FFU)/mL for DENV-3 and DENV-4

^b^RNAemia was measured by DENV-specific real-time RT-PCR and expressed as log10 genome equivalents (ge)/mL

^c^Times to detection are expressed as the averaged day post-inoculation for viremia or RNAemia onset from macaques inoculated with the same viral isolate

^d^Durations are expressed as the mean number of days with detectable viremia or RNAemia from macaques inoculated with the same viral isolate

^e^Peak titers are expressed as the averaged peak titer for viremia or RNAemia from macaques inoculated with the same viral isolate

^f^Detected in only 1 out of the 2 inoculated macaques; nd, not detected.

### DENV-neutralizing antibody responses detected after infection

**Fig 2:**
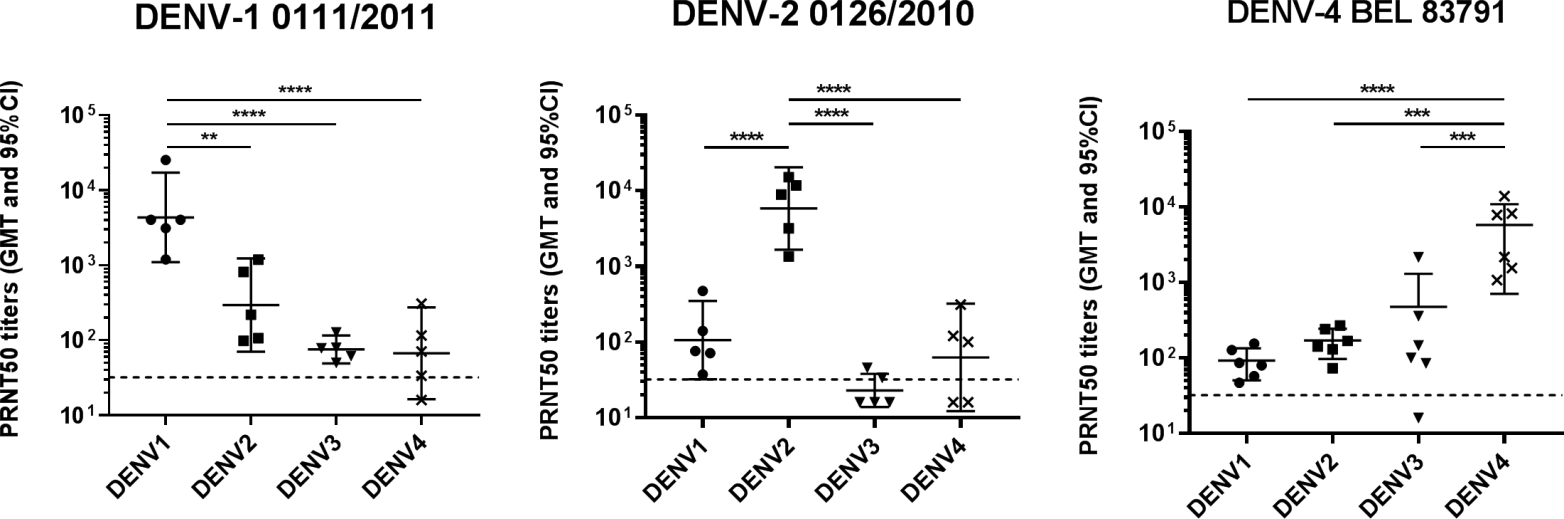
DENV-neutralizing antibody responses detected after infection with DENV-1 0111/2011, DENV-2 0126/2010 or DENV-4 BEL 83791. Sera collected 2 weeks after DENV inoculation were tested by plaque-reduction neutralization test (PRNT) for their neutralizing activity against DENV-1 60305, DENV-2 44/2, DENV-3 16562 and DENV-4 TVP360. The individual serum titers associated with 50% reduction in plaque counts (PRNT50), geometric mean titers (GMT) and 95% confidence intervals (CI) are shown. Dashed lines indicate the limit of detection. In the absence of detection of neutralizing activity, the corresponding sample was assigned an arbitrary titer corresponding to half the limit of detection. *P*-values were calculated using the Tukey’s multiple comparisons test: **, *p*<0.01; ***, *p*<0.001; ****, *p*<0.0001.

To characterize the elicited DENV-neutralizing antibody responses, sera collected 2 weeks after viral inoculation were tested for their neutralizing activity by PRNT. Fig 2 shows the PRNT50 titers measured after infection with DENV-1 0111/2011, DENV-2 0126/2010 or DENV-4 BEL 83791. High PRNT50 titers, ranking from ~1000 to 10000, were detected against the homologous serotypes, indirectly corroborating the high-level replication of these isolates. Cross-serotype neutralizing antibody responses were also detected, with the highest and broadest levels of cross-reactivity observed after infection with either DENV-1 0111/2011 or DENV-4 BEL 83791. DENV-2 0126/2010 elicited an antibody response that was mainly homologous to the infecting serotype while the cross-neutralizing antibody titers were not detected or close to the detection threshold. Besides the three selected isolates, all tested DENV isolates except DENV-3 ROR 6210 elicited broad DENV-neutralizing antibody responses that were strongly biased towards the homologous serotype (data not shown). This demonstrated that, except for DENV-3 ROR 6210, all inoculated isolates elicited a virus-specific adaptive immune response even when viral replication was low or undetectable.

### Serum cytokine profile associated with infection with DENV-1 0111/2011, DENV-2 0126/2010 or DENV-4 BEL 83791

**Fig 3:**
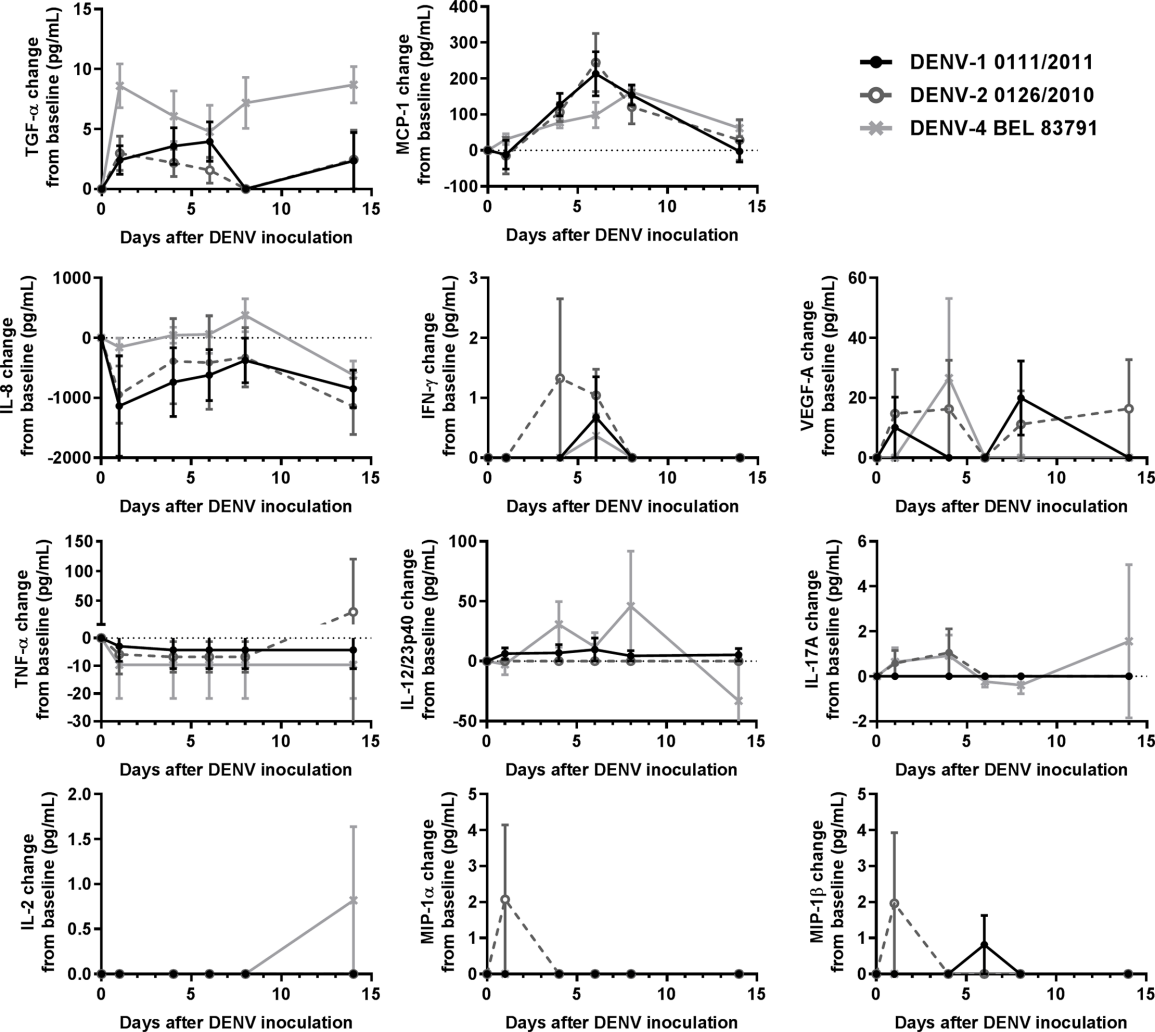
Serum cytokine profiles observed after infection with DENV-1 0111/2011, DENV-2 0126/2010 or DENV-4 BEL 83791. Sera collected before (baseline) and at days 1, 4, 6, 8 and 14 after DENV inoculation were tested, in duplicate, for their concentration in the indicated cytokines. Results were expressed as pg/mL. When no signal was detected, the corresponding sample was assigned the arbitrary value of half the limit of detection for the corresponding cytokine. Shown are the mean changes from baseline and SEM from 5 (DENV-1 0111/2011 and DENV-2 0126/2010) and 6 (DENV-4 BEL 83791) animals.

To characterize the cytokine profile associated with DENV infection in rhesus macaques, sera collected before and 1, 4, 6, 8 and 14 days after infection were tested, using a multiplexed microbead-based cytokine assay, for their concentration in soluble IFN-γ, IL-1β, IL-2, IL-6, IL-8, IL-10, IL-12/23, IL-17A, IL-18, MIP-1α, MIP-1ß, TNF-α, TGF-α, VEGF-A and MCP-1. Results obtained after infection with DENV-1 0111/2011, DENV-2 0126/2010 or DENV-4 BEL 83791 are shown in Fig 3. No IL-1β, IL-6, IL-10 or IL-18 responses were detected (data not shown) and IL-2, MIP-1α and MIP-1ß were detected only in one macaque infected with DENV-4 BEL 83791 and, for MIP-1ß only, in one macaque infected with DENV-1 0111/2011. In contrast, increased MCP-1, TGF-α and IFN-γ levels, and transiently decreased IL-8 levels were observed after infection, irrespective of the DENV serotype. The increased MCP-1 levels were statistically significant at days 4, 6 and 8 post-infection for all three isolates (*p*≤0.0001 for DENV-1 0111/2011 and DENV-2 0126/2010 and *p*≤0.05 for DENV-4 BEL 83791). The increased TGF-α levels, observed at all tested time points except at day 8 post-inoculation with DENV-1 0111/2011 or DENV-2 0126/2010, were statistically significant for both DENV-1 0111/2011 (*p*≤0.05 at days 4 and 6 post-infection) and DENV-4 BEL 83791 (*p*≤0.005 at all tested time points post-infection). The increased IFN-γ levels, at day 6 post-infection with DENV-1 0111/2011 and DENV-4 BEL 83791 and at days 4 and 6 with DENV-2 0126/2010, were statistically significant for DENV-2 0126/2010 (*p*≤0.01 at both days 4 and 6 post-infection). The decreased IL-8 levels were statistically significant at days 1 and 14 (*p*≤0.05). Analysis of the kinetics of the cytokine responses in relation to the viremia showed that maximum TGF-α levels were detected either before or concurrent with the day of peak viremia, whereas the maximum MCP-1 and INF-γ levels occurred after the peak of viremia (data not shown). Moreover, although not statistically significant, increased VEGF-A levels were detected after infection with all three DENV isolates, with maximum levels observed both before and after the viremia peak. Finally, slight and not statistically significant trends for increased IL-17A levels and decreased TNF-α levels were also observed.

## Discussion

This study describes the identification of three recent and minimally passaged DENV isolates from serotypes 1, 2 and 4 which, after inoculation into rhesus macaques, induced robust viremia/RNAemia, strong DENV-neutralizing antibody responses, and a modification of the serum cytokine profile which shared similarities with those associated with dengue in humans. No DENV infection-associated clinical symptom or abnormal behavior was observed here. In addition, post-mortem analysis was performed for all macaques used in this study. Tissue samples, collected both from the central nervous system and the extraneural organs, including salivary glands, heart, lungs, liver, pancreas, duodenum, stomach, spleen, small and large bowel, kidneys and lymph nodes (axillar, inguinal and mesenteric), were stained with hematoxylin and eosin and further analyzed for microscopic lesions. None of the thirty inoculated rhesus monkeys developed any histological lesion (data not shown).

Low-passage DENV clinical isolates were previously shown to be infectious in marmosets and tamarins [31–33] and one study reported infectivity of so-called wild DENV isolates in rhesus macaques [34]. However, the DENV isolates used in this study had been subjected to more than ten sequential *in vitro* passages in the same cell system and might thus have lost their wild-type phenotype. In contrast, the isolates described here were passaged in cell culture only three times for DENV-1 0111/2011 and DENV-2 0126/2010 and four times for DENV-4 BEL 83791, with no more than two sequential passages within the same cell system.

A meta-analysis of viremia kinetics during primary DENV infection in rhesus macaques, based on 36 published studies including 466 animals, reported median times to detectable viremia of 3.32 days, 2.63 days and 3.23 days for DENV-1, DENV-2 and DENV-4, respectively [27]. With mean times to viremia of 2 days for DENV-1 0111/2011 and 1 day for DENV-2 0126/2010 and DENV-4 BEL 83791 in the current study, these isolates are among the 5–25% of isolates reported to be associated with the shortest times to detectable viremia. The same meta-analysis also revealed median viremia durations in rhesus macaques of 4.67 days, 5.13 days and 3.13 days for DENV-1, DENV-2 and DENV-4, respectively [27]. With mean viremia durations of 6.4 days for DENV-1 0111/2011, 6.2 days for DENV-2 0126/2010 and 4.17 days for DENV-4 BEL 83791 observed in the current study, these isolates were associated with longer viremia durations than those reported for most of the isolates previously tested in this model. Finally, the viremia peak titers detected after inoculation with DENV-1 0111/2011, DENV-2 0126/2010 and DENV-4 BEL 83791 (10^2.73^, 10^3.50^ and 10^2.36^ PFU/mL, respectively) fell within the ranges of the highest titers previously described in rhesus macaques [5]. Altogether, the three isolates described here induced viremia of longer duration and high magnitude relative to previously reported viremia kinetics in this model, opening the door to a dengue macaque model better mirroring the DENV viremia kinetics observed in human dengue virus challenge studies [35].

The selected DENV isolates elicited strong homotypic DENV-neutralizing antibody responses and, although of lower magnitude (particularly with DENV-2 0126/2010), also heterotypic responses (Fig 2). This is consistent with observations made after primary DENV infection both in humans [36,37] and macaques [38–40]. While DENV-2 0126/2010 elicited here the highest homotypic response, a serotype 4 isolate had been previously associated, in macaque, with the highest homotypic antibody responses [38]. We speculate that the balance of elicited homotypic *versus* heterotypic responses might be strain- rather than serotype-specific.

In our study, each of the three selected isolates induced a drastic increase in MCP-1 levels. MCP-1 drives recruitment of monocytes/macrophages and memory T cells and is secreted by monocytes [41], the main DENV target cells. MCP-1 has been widely documented as being increased during dengue fever and severe dengue in humans [1,19,20,23] and might directly induce alterations of the vascular endothelium [42]. Interestingly, increased serum MCP-1 levels had been previously reported in rhesus macaques after DENV infection [39], further confirming its association with DENV infection in this model. A slight but significant increase in IFN-γ levels was also observed, corroborating what has been reported both in humans [1,20,23] and once in rhesus macaques [39]. In addition, although not statistically significant, increased VEGF-A levels were observed after infection with each of the 3 selected isolates, which has not been previously reported in macaques. VEGF-A, the most potent permeability-enhancing cytokine known, has been widely reported as showing elevated levels in patients experiencing dengue fever and severe dengue and is considered as a potential marker of disease severity [1,20]. We also observed a transient and early decrease in IL-8 levels after DENV infection, which had not been previously reported in rhesus macaques [39,43], possibly because it was not assessed at early time points after infection. However and corroborating our results, a transient and early decrease in IL-8 transcription was reported after DENV infection in cynomolgus macaques [40]. Interestingly, while IL-8 was shown to be increased during severe dengue in humans [20,44,45], it was also reported once as being decreased in patients with classical/mild dengue fever [44]. We speculate that the early decreased IL-8 levels after DENV infection in macaques might, at least partially, explain the lack of dengue disease in this model. Finally, we observed significantly increased levels of TGF-α which has, to our knowledge, never been reported as being associated with DENV infection, neither in humans nor in macaques. TGF-α, which belongs to the epidermal growth factor family, is expressed in the liver where it can stimulate hepatocyte proliferation, and was reported as being closely related to the severity of liver dysfunction [46]. Given that hepatocytes are important targets for DENV and that liver dysfunction is frequently associated with dengue [7,37], it is not surprising to detect increased TGF-α levels during DENV infection.

In conclusion, we identified three recent and minimally passaged DENV isolates that induced a robust infection in rhesus macaques. In line with reported features of dengue fever in humans, infection with these isolates was also associated with increased levels of MCP-1, IFN-γ and VEGF-A and a transiently decreased IL-8 level. These results open the door to an improved dengue macaque model.

## Supporting information

S1 File Viremia and RNAemia raw data. (XLSX)

S2 File PRNT raw data. (XLSX)

S3 File Cytokine raw data. (XLSX)

## References

[1] Guzman MG, Harris E. Dengue. Lancet. 2015; 385: 453–465. doi: 10.1016/S0140-6736(14)60572-9 25230594 10.1016/S0140-6736(14)60572-9

[2] Stanaway JD, Shepard DS, Undurraga EA, Halasa YA, Coffeng LE, Brady OJ, et al The global burden of dengue: an analysis from the Global Burden of Disease Study 2013. Lancet Infect Dis. 2016; 16: 712–723. doi: 10.1016/S1473-3099(16)00026-8 26874619 10.1016/S1473-3099(16)00026-8 PMC5012511

[3] Capeding MR, Tran NH, Hadinegoro SR, Ismail HI, Chotpitayasunondh T, Chua MN, et al Clinical efficacy and safety of a novel tetravalent dengue vaccine in healthy children in Asia: a phase 3, randomised, observer-masked, placebo-controlled trial. Lancet. 2014; 384: 1358–1365. doi: 10.1016/S0140-6736(14)61060-6 25018116 10.1016/S0140-6736(14)61060-6

[4] Villar L, Dayan GH, Arredondo-Garcia JL, Rivera DM, Cunha R, Deseda C, et al Efficacy of a tetravalent dengue vaccine in children in Latin America. N Engl J Med. 2015; 372: 113–123. doi: 10.1056/NEJMoa1411037 25365753 10.1056/NEJMoa1411037

[5] Clark KB, Onlamoon N, Hsiao HM, Perng GC, Villinger F. Can non-human primates serve as models for investigating dengue disease pathogenesis? Front Microbiol. 2013; 4: 305 doi: 10.3389/fmicb.2013.00305 24130557 10.3389/fmicb.2013.00305 PMC3795305

[6] Sariol CA, White LJ. Utility, limitations, and future of non-human primates for dengue research and vaccine development. Front Immunol. 2014; 5: 452 doi: 10.3389/fimmu.2014.00452 25309540 10.3389/fimmu.2014.00452 PMC4174039

[7] St John AL, Abraham SN, Gubler DJ. Barriers to preclinical investigations of anti-dengue immunity and dengue pathogenesis. Nat Rev Microbiol. 2013; 11: 420–426. doi: 10.1038/nrmicro3030 23652323 10.1038/nrmicro3030

[8] Blaney JE Jr., Matro JM, Murphy BR, Whitehead SS. Recombinant, live-attenuated tetravalent dengue virus vaccine formulations induce a balanced, broad, and protective neutralizing antibody response against each of the four serotypes in rhesus monkeys. J Virol. 2005; 79: 5516–5528. doi: 10.1128/JVI.79.9.5516-5528.2005 15827166 10.1128/JVI.79.9.5516-5528.2005 PMC1082773

[9] Clements DE, Coller BA, Lieberman MM, Ogata S, Wang G, Harada KE, et al Development of a recombinant tetravalent dengue virus vaccine: immunogenicity and efficacy studies in mice and monkeys. Vaccine. 2010; 28: 2705–2715. doi: 10.1016/j.vaccine.2010.01.022 20097152 10.1016/j.vaccine.2010.01.022 PMC2837772

[10] Fernandez S, Thomas SJ, De La Barrera R, Im-Erbsin R, Jarman RG, Baras B, et al An adjuvanted, tetravalent dengue virus purified inactivated vaccine candidate induces long-lasting and protective antibody responses against dengue challenge in rhesus macaques. Am J Trop Med Hyg. 2015; 92: 698–708. doi: 10.4269/ajtmh.14-0268 25646261 10.4269/ajtmh.14-0268 PMC4385761

[11] Guirakhoo F, Pugachev K, Zhang Z, Myers G, Levenbook I, Draper K, et al Safety and efficacy of chimeric yellow Fever-dengue virus tetravalent vaccine formulations in nonhuman primates. J Virol. 2004; 78: 4761–4775. doi: 10.1128/JVI.78.9.4761-4775.2004 15078958 10.1128/JVI.78.9.4761-4775.2004 PMC387722

[12] Koraka P, Benton S, van AG, Stittelaar KJ, Osterhaus AD. Efficacy of a live attenuated tetravalent candidate dengue vaccine in naive and previously infected cynomolgus macaques. Vaccine. 2007; 25: 5409–5416. doi: 10.1016/j.vaccine.2007.04.079 17560694 10.1016/j.vaccine.2007.04.079

[13] Osorio JE, Brewoo JN, Silengo SJ, Arguello J, Moldovan IR, Tary-Lehmann M, et al Efficacy of a tetravalent chimeric dengue vaccine (DENVax) in Cynomolgus macaques. Am J Trop Med Hyg. 2011; 84: 978–987. doi: 10.4269/ajtmh.2011.10-0592 21633037 10.4269/ajtmh.2011.10-0592 PMC3110349

[14] Gubler DJ. Dengue and dengue hemorrhagic fever. Clin Microbiol Rev. 1998; 11: 480–496. 9665979 10.1128/cmr.11.3.480 PMC88892

[15] Vaughn DW, Green S, Kalayanarooj S, Innis BL, Nimmannitya S, Suntayakorn S, et al Dengue viremia titer, antibody response pattern, and virus serotype correlate with disease severity. J Infect Dis. 2000; 181: 2–9. doi: 10.1086/315215 10608744 10.1086/315215

[16] Coffey LL, Vasilakis N, Brault AC, Powers AM, Tripet F, Weaver SC. Arbovirus evolution in vivo is constrained by host alternation. Proc Natl Acad Sci U S A. 2008; 105: 6970–6975. doi: 10.1073/pnas.0712130105 18458341 10.1073/pnas.0712130105 PMC2383930

[17] Weaver SC, Rico-Hesse R, Scott TW. Genetic diversity and slow rates of evolution in New World alphaviruses. Curr Top Microbiol Immunol. 1992; 176: 99–117. 1318187 10.1007/978-3-642-77011-1_7

[18] Greene IP, Wang E, Deardorff ER, Milleron R, Domingo E, Weaver SC. Effect of alternating passage on adaptation of sindbis virus to vertebrate and invertebrate cells. J Virol. 2005; 79: 14253–14260. doi: 10.1128/JVI.79.22.14253-14260.2005 16254360 10.1128/JVI.79.22.14253-14260.2005 PMC1280187

[19] Rothman AL. Immunity to dengue virus: a tale of original antigenic sin and tropical cytokine storms. Nat Rev Immunol. 2011; 11: 532–543. doi: 10.1038/nri3014 21760609 10.1038/nri3014

[20] Srikiatkhachorn A, Mathew A, Rothman AL. Immune-mediated cytokine storm and its role in severe dengue. Semin Immunopathol. 2017; 39: 563–574. doi: 10.1007/s00281-017-0625-1 28401256 10.1007/s00281-017-0625-1 PMC5496927

[21] Chen LC, Lei HY, Liu CC, Shiesh SC, Chen SH, Liu HS, et al Correlation of serum levels of macrophage migration inhibitory factor with disease severity and clinical outcome in dengue patients. Am J Trop Med Hyg. 2006; 74: 142–147. 16407359

[22] Dejnirattisai W, Duangchinda T, Lin CL, Vasanawathana S, Jones M, Jacobs M, et al A complex interplay among virus, dendritic cells, T cells, and cytokines in dengue virus infections. J Immunol. 2008; 181: 5865–5874. 18941175 10.4049/jimmunol.181.9.5865

[23] Green S, Rothman A. Immunopathological mechanisms in dengue and dengue hemorrhagic fever. Curr Opin Infect Dis. 2006; 19: 429–436. doi: 10.1097/01.qco.0000244047.31135.fa 16940865 10.1097/01.qco.0000244047.31135.fa

[24] Her Z, Kam YW, Gan VC, Lee B, Thein TL, Tan JJ, et al Severity of Plasma Leakage Is Associated With High Levels of Interferon gamma-Inducible Protein 10, Hepatocyte Growth Factor, Matrix Metalloproteinase 2 (MMP-2), and MMP-9 During Dengue Virus Infection. J Infect Dis. 2017; 215: 42–51. doi: 10.1093/infdis/jiw494 28077582 10.1093/infdis/jiw494

[25] Kwissa M, Nakaya HI, Onlamoon N, Wrammert J, Villinger F, Perng GC, et al Dengue virus infection induces expansion of a CD14(+)CD16(+) monocyte population that stimulates plasmablast differentiation. Cell Host Microbe. 2014; 16: 115–127. doi: 10.1016/j.chom.2014.06.001 24981333 10.1016/j.chom.2014.06.001 PMC4116428

[26] Lee YH, Leong WY, Wilder-Smith A. Markers of dengue severity: a systematic review of cytokines and chemokines. J Gen Virol. 2016; 97: 3103–3119. doi: 10.1099/jgv.0.000637 27902364 10.1099/jgv.0.000637

[27] Althouse BM, Durbin AP, Hanley KA, Halstead SB, Weaver SC, Cummings DA. Viral kinetics of primary dengue virus infection in non-human primates: a systematic review and individual pooled analysis. Virology. 2014; 452–453: 237–246. 10.1016/j.virol.2014.01.015 PMC4578724 24606701

[28] Rico-Hesse R. Microevolution and virulence of dengue viruses. Adv Virus Res. 2003; 59: 315–341. 14696333 10.1016/s0065-3527(03)59009-1 PMC3045824

[29] Miagostovich MP, Sequeira PC, Dos Santos FB, Maia A, Nogueira RM, Schatzmayr HG, et al Molecular typing of dengue virus type 2 in Brazil. Rev Inst Med Trop Sao Paulo. 2003; 45: 17–21. 10.1590/s0036-46652003000100004 12751317

[30] Gubler DJ, Rosen L. A simple technique for demonstrating transmission of dengue virus by mosquitoes without the use of vertebrate hosts. Am J Trop Med Hyg. 1976; 25: 146–150. 3980 10.4269/ajtmh.1976.25.146

[31] Ferreira MS, de Castro PH, Silva GA, Casseb SM, Dias Junior AG, Rodrigues SG, et al Callithrix penicillata: a feasible experimental model for dengue virus infection. Immunol Lett. 2014; 158: 126–133. doi: 10.1016/j.imlet.2013.12.008 24361035 10.1016/j.imlet.2013.12.008

[32] Omatsu T, Moi ML, Hirayama T, Takasaki T, Nakamura S, Tajima S, et al Common marmoset (Callithrix jacchus) as a primate model of dengue virus infection: development of high levels of viraemia and demonstration of protective immunity. J Gen Virol. 2011; 92: 2272–2280. doi: 10.1099/vir.0.031229-0 21697346 10.1099/vir.0.031229-0

[33] Yoshida T, Omatsu T, Saito A, Katakai Y, Iwasaki Y, Iijima S, et al CD16(+) natural killer cells play a limited role against primary dengue virus infection in tamarins. Arch Virol. 2012; 157: 363–368. doi: 10.1007/s00705-011-1178-6 22139354 10.1007/s00705-011-1178-6

[34] Freire M, Marchevsky R, Almeida L, Yamamura A, Caride E, Brindeiro P, et al Wild dengue virus types 1, 2 and 3 viremia in rhesus monkeys. Mem Inst Oswaldo Cruz. 2007; 102: 203–208. 17426886 10.1590/s0074-02762007005000011

[35] Larsen CP, Whitehead SS, Durbin AP. Dengue human infection models to advance dengue vaccine development. Vaccine. 2015; 33: 7075–7082. doi: 10.1016/j.vaccine.2015.09.052 26424605 10.1016/j.vaccine.2015.09.052

[36] Endy TP. Human immune responses to dengue virus infection: lessons learned from prospective cohort studies. Front Immunol. 2014; 5: 183 doi: 10.3389/fimmu.2014.00183 24795725 10.3389/fimmu.2014.00183 PMC4006038

[37] Murphy BR, Whitehead SS. Immune response to dengue virus and prospects for a vaccine. Annu Rev Immunol. 2011; 29: 587–619. doi: 10.1146/annurev-immunol-031210-101315 21219187 10.1146/annurev-immunol-031210-101315

[38] Halstead SB, Shotwell H, Casals J. Studies on the pathogenesis of dengue infection in monkeys. I. Clinical laboratory responses to primary infection. J Infect Dis. 1973; 128: 7–14. 4198027 10.1093/infdis/128.1.7

[39] Hickey AC, Koster JA, Thalmann CM, Hardcastle K, Tio PH, Cardosa MJ, et al Serotype-specific host responses in rhesus macaques after primary dengue challenge. Am J Trop Med Hyg. 2013; 89: 1043–1057. doi: 10.4269/ajtmh.13-0145 24062475 10.4269/ajtmh.13-0145 PMC3854881

[40] Koraka P, Benton S, van AG, Stittelaar KJ, Osterhaus AD. Characterization of humoral and cellular immune responses in cynomolgus macaques upon primary and subsequent heterologous infections with dengue viruses. Microbes Infect. 2007; 9: 940–946. doi: 10.1016/j.micinf.2007.03.012 17544804 10.1016/j.micinf.2007.03.012

[41] Deshmane SL, Kremlev S, Amini S, Sawaya BE. Monocyte chemoattractant protein-1 (MCP-1): an overview. J Interferon Cytokine Res. 2009; 29: 313–326. doi: 10.1089/jir.2008.0027 19441883 10.1089/jir.2008.0027 PMC2755091

[42] Appanna R, Wang SM, Ponnampalavanar SA, Lum LC, Sekaran SD. Cytokine factors present in dengue patient sera induces alterations of junctional proteins in human endothelial cells. Am J Trop Med Hyg. 2012; 87: 936–942. doi: 10.4269/ajtmh.2012.11-0606 22987650 10.4269/ajtmh.2012.11-0606 PMC3516272

[43] Sariol CA, Munoz-Jordan JL, Abel K, Rosado LC, Pantoja P, Giavedoni L, et al Transcriptional activation of interferon-stimulated genes but not of cytokine genes after primary infection of rhesus macaques with dengue virus type 1. Clin Vaccine Immunol. 2007; 14: 756–766. doi: 10.1128/CVI.00052-07 17428947 10.1128/CVI.00052-07 PMC1951081

[44] Pandey N, Jain A, Garg RK, Kumar R, Agrawal OP, Lakshmana Rao PV. Serum levels of IL-8, IFNgamma, IL-10, and TGF beta and their gene expression levels in severe and non-severe cases of dengue virus infection. Arch Virol. 2015; 160: 1463–1475. doi: 10.1007/s00705-015-2410-6 25860648 10.1007/s00705-015-2410-6

[45] Raghupathy R, Chaturvedi UC, Al-Sayer H, Elbishbishi EA, Agarwal R, Nagar R, et al Elevated levels of IL-8 in dengue hemorrhagic fever. J Med Virol. 1998; 56: 280–285. 9783699 10.1002/(sici)1096-9071(199811)56:3<280::aid-jmv18>3.0.co;2-i

[46] Badawy AA, El-Hindawi A, Hammam O, Moussa M, Gabal S, Said N. Impact of epidermal growth factor receptor and transforming growth factor-alpha on hepatitis C virus-induced hepatocarcinogenesis. APMIS. 2015; 123: 823–831. doi: 10.1111/apm.12431 26279457 10.1111/apm.12431

